# Byzantine—Early Islamic resource management detected through micro-geoarchaeological investigations of trash mounds (Negev, Israel)

**DOI:** 10.1371/journal.pone.0239227

**Published:** 2020-10-14

**Authors:** Don H. Butler, Zachary C. Dunseth, Yotam Tepper, Tali Erickson-Gini, Guy Bar-Oz, Ruth Shahack-Gross

**Affiliations:** 1 Laboratory for Sedimentary Archaeology, Department of Maritime Civilizations, Recanati Institute of Maritime Studies, Leon H. Charney School of Marine Sciences, University of Haifa, Haifa, Israel; 2 Jacob M. Alkow Department of Archaeology and Ancient Near Eastern Cultures, Tel Aviv University, Tel Aviv, Israel; 3 Zinman Institute of Archaeology, University of Haifa, Haifa, Israel; 4 Israel Antiquities Authority, Tel Aviv, Israel; 5 Archaeological Division, Israel Antiquities Authority, Omer, Israel; Institute for Anthropological Research, CROATIA

## Abstract

Sustainable resource management is of central importance among agrarian societies in marginal drylands. In the Negev Desert, Israel, research on agropastoral resource management during Late Antiquity emphasizes intramural settlement contexts and landscape features. The importance of hinterland trash deposits as diachronic archives of resource use and disposal has been overlooked until recently. Without these data, assessments of community-scale responses to societal, economic, and environmental disruption and reconfiguration remain incomplete. In this study, micro-geoarchaeological investigations were conducted on trash mound features at the Byzantine—Early Islamic sites of Shivta, Elusa, and Nesanna to track spatiotemporal trends in the use and disposal of critical agropastoral resources. Refuse derived sediment deposits were characterized using stratigraphy, micro-remains (i.e., livestock dung spherulites, wood ash pseudomorphs, and plant phytoliths), and mineralogy by Fourier transform infrared spectroscopy. Our investigations detected a turning point in the management of herbivore livestock dung, a vital resource in the Negev. We propose that the scarcity of raw dung proxies in the studied deposits relates to the use of this resource as fuel and agricultural fertilizer. Refuse deposits contained dung ash, indicating the widespread use of dung as a sustainable fuel. Sharply contrasting this, raw dung was dumped and incinerated outside the village of Nessana. We discuss how this local shift in dung management corresponds with a growing emphasis on sedentised herding spurred by newly pressed taxation and declining market-oriented agriculture. Our work is among the first to deal with the role of waste management and its significance to economic strategies and urban development during the late Roman Imperial Period and Late Antiquity. The findings contribute to highlighting top-down societal and economic pressures, rather than environmental degradation, as key factors involved in the ruralisation of the Negev agricultural heartland toward the close of Late Antiquity.

## Introduction

Issues surrounding resource and waste management figure prominently within debates concerning the causes and consequences of societal and environmental transformations through time [1–7]. This topic, one central to opening the conceptual, empirical, and pragmatic scope of the Anthropocene, benefits from archaeological research into the factors shaping socio-ecological dynamics at multiple nested spatialities and tempos. Micro-geoarchaeological research in urban and hinterland settings contributes to clarifying aspects of these processes that are often imperceptible in records of material culture [8–10]. Studies of anthrosediments deposited and formed through trash disposal and management are specifically providing fresh insight into changing economic systems, (un)sustainable resource-use, human-plant-animal networks, niche construction, urban renewal/decay, and settlement strategies [10–16].

Trash disposal locations, however, are often distorted or erased at cities, towns, and villages that have witnessed uninterrupted occupation and renewal throughout the past and into the modern era. Signposts for economic fluorescence and erosion are difficult to follow through the dense palimpsests formed at long-lived communities, such as those growing throughout the Roman Imperial Period, Late Antiquity, and beyond [17, 18]. Without these data on refuse disposal and resource management, our assessments of how different societies and communities contribute and respond to disruptions in resource structures remain fragmentary. These assessments are needed to develop holistic strategies for recognizing early signs of resource management issues such as overproduction/consumption, ecological overshoot, and depreciated surplus in the past, present, and near future [6, 7]. This is particularly important in marginal and threatened environments such as drylands.

Contrasting the above unending city scenario, the once prosperous Byzantine and Early Islamic (Umayyad) farm villages and central town (Elusa) of the Negev Desert, Israel, were in decline and ultimately abandoned between the 7^th^ and 10^th^ centuries CE [19–24]. The trash mounds flanking their walls remain prominent on the landscape today, never completely overwritten by urban sprawl. They now present exceptional opportunities to examine relationships between resource management (i.e., use and disposal), economics, social organization, and ecological transformations. Diverse approaches to conceptualizing and operationalizing these features as gauges for long-term societal change and human-ecodynamics are developing [11, 22, 23]. Here, we contribute to this growing area of research from a micro-geoarchaeological perspective. We use micro-sedimentary evidence from hinterland trash mounds to investigate spatiotemporal trends in resource management and agropastoral dynamics that are not accessible in records of material culture recovered from intramural village contexts. These data will contribute to debates surrounding the causes and consequences of disruption and reconfiguration in agropastoral niches toward the close of Late Antiquity [see, for example, 11, 18–23].

**Fig 1:**
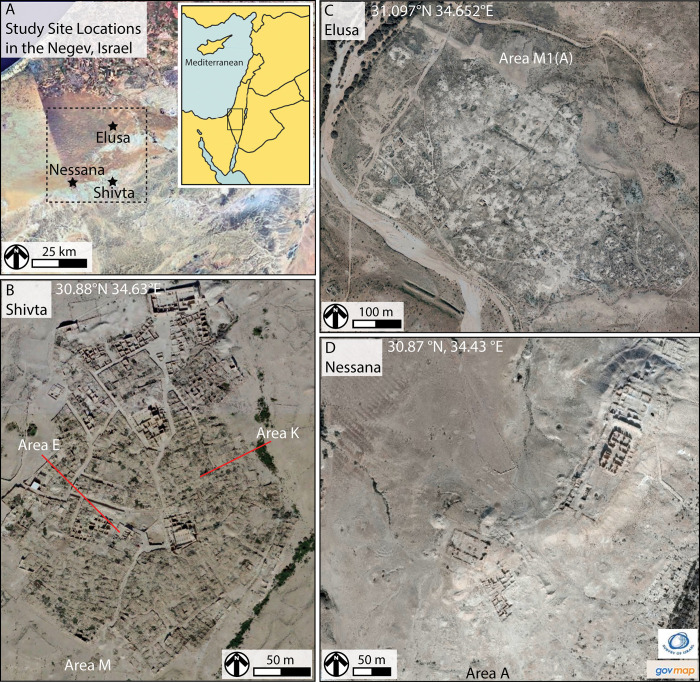
Study locations. (A) Overview of the Negev region showing the three archaeological sites investigated in this study. The approximate perimeter of the urban/agriculture heartland is outlined. (B) Aerial view of Shivta with location of the excavated Area M Byzantine trash mound, as well as Areas E and K where small Early Islamic middens are located. (C) Aerial view of Elusa with location of the excavated Area M1(A) Byzantine trash mound. (D) Aerial view of Nessana with location of the excavated Area A trash mound, where Byzantine trash is overlain by Early Islamic trash. Orthophoto images reprinted under a CC BY license, with permission from The Survey of Israel (gov map), 2019.

Our study focuses on trash mound sediments from three of the six major Negev settlements: the UNESCO World Heritage Sites of Shivta and Elusa, as well as the long-lived village of Nessana (Fig 1). These sites were selected for study because of their well understood chronologies and economic systems, as well as the presence of stratified trash mounds spanning the Byzantine—Early Islamic periods (c. 4^th^ - 10^th^ century CE) [11, 22, 23]. The investigation aims to 1) characterize the sediment deposits comprising hinterland trash mounds and classify the resource/refuse types represented, 2) track changes in the use and disposal of agropastoral resources through time and between villages, and 3), contextualize these findings within newly developing understandings of the rise and fall of Negev agropastoral systems during Late Antiquity. We show how changes in the management of critical dryland resources, specifically livestock dung, are registered in the sedimentary archives comprising the studied trash mounds. The work underscores the value of micro-sedimentary archives in classical studies aiming to track long-term societal change and human-environment interactions in urban settings. Our findings provide much-needed new insight into community specific responses to social and economic transformations in the Negev during a pivotal time in its history–during the collapse of market-oriented agriculture and ruralisation of the urban heartland near the end of the first millennium CE.

### Environmental conditions in the Negev

The study area sits at elevations ranging from 500–1,000 m above sea level (Fig 1). Bedrock is primarily marine carbonate rocks covered by thick loess deposits (aeolian sediment) [25]. The region is currently arid, yet precipitation is variable, with annual lows of 30 mm and highs reaching 180 mm [26]. The mean annual temperature is 19°C, with average monthly temperatures ranging from 8°C in January to 26°C in August [27, 28]. Slowly growing Saharo-Arabian vegetation is sparsely distributed [29, 30]. Regional records from the Dead Sea (sediment cores) and Soreq Cave (speleothems) suggest that arid conditions (interspersed with short precipitation increases) have prevailed in the Negev Highlands over the past 5,000 years [31–35]. Caves in the Highlands have not formed speleothems over the past 86,000 years, indicating long-term annual precipitation levels not exceeding 200–275 mm [36]. Recent stable isotope analyses of sheep and goat teeth from the Negev also indicate unchanging forage quality through the Byzantine and Early Islamic periods, which is suggestive of climatic stability [37]. Lithosols, reg soils, and calcic soils have been forming across the region since at least the end of the Pleistocene [26, 38]. Calcic soil formation indicates annual precipitation below 200 mm [38]. Pore spaces in these soil types are often filled with precipitating carbonates. This reduces water holding capacity, movement, and retention. It also limits plant root proliferation. Negev soils/sediments typically have low organic carbon (e.g., 0.15–0.57%) and low nitrogen (indicated by low nitrates, e.g., 6.35–6.78 mg/kg) [39, 40]. These factors constrain above ground biomass production.

The precipitation levels and soil/sediment quality of the region were unfavorable for high yield direct rain-fed agriculture during Late Antiquity [26]. Estimated precipitation was below the optimal quantity required to cultivate large crops of wheat, barley, and fruit (i.e., ~ 250–400 mm annually) [41]. Despite suboptimal conditions, upward of 30,000 acres of terraced fields, orchards, and vineyards were established during the Byzantine period [20, 42]. Such large-scale agriculture could only be sustained through extensive human transformation and maintenance of the landscape [20, 26, 41, 43, 44]. Farming was successful under harsh environmental conditions because of innovations in environmental niche engineering, specifically the development of landscape terracing, runoff water/sediment harvesting systems, and sustainable fertilizer production [19, 20, 24, 45–56].

### A micro-geoarchaeological approach to trash mound deposits and resource management

A highly resilient runoff agriculture niche provided the economic backbone supporting Byzantine and Early Islamic (c. 4^th^ - 10^th^ century CE) communities in the environmentally marginal Negev borderlands [11, 19, 20, 23, 41–46]. The success of agriculture was conditioned by people’s control over two key limiting factors in the region: water availability and soil quality. Owing to the region’s prevailing aridity, weak pedogenesis, and pervasive erosion, the productivity of this system relied on the transformation of desert sediments into arable lands through the installation of water and sediment harvesting systems [19, 20, 24, 45–56; see 23, 24, 45 for an alternative view].

Farmland improvement and sustainability were forefront concerns among Negev farmers [20, 24, 45, and references therein]. We therefore expect that farmers used fertilizers to maximize productivity. The installation of dovecotes at the Byzantine farming villages of Shivta and Sa‘adon strongly implies the use of animal wastes as fertilizers [24, 45; see 57, 58 for information on Transjordan use]. Ovicaprine (sheep/goat) herding was also an important sector of the economy [11, 37, 44]. It stands to reason that the wide availability of ovicaprine dung made this material a valuable fertilizer as well. Recent investigations of sediments from terraced wadi fields at Horvat Haluqim, for example, provide new evidence suggesting the use of domestic wastes and herbivore dung as agricultural fertilizers [27]. Dung fertilizers would contribute to improving the macronutrient status, structure, porosity, water holding capacity, and cation exchange capacity of agricultural soils [27, 59, 60]. Runoff harvesting systems would have supplied the water required to raise the effectiveness of added dung fertilizers. Along with this, dung was also used as a fuel resource because of the scarcity and slow growth of local woody vegetation [15, 29, 61].

We hypothesize that livestock dung fertilizers and sustainable dung resource management were important components of Negev agricultural niches. The use and disposal of dung resources and the scope of agropastoral systems are therefore expected to covary. Changes in the use/disposal of dung resources, and agricultural and pastoral economics, are also expected to be registered in the compositions of the anthrosediments comprising trash mounds. Within a large-scale market-oriented agriculture system, raw dung would have been valued as fertilizer and fuel. We therefore expect trash dumps to contain small quantities of raw dung, if any. The dung present in dumps should primarily appear in the form of ash, representing the disposal of spent domestic fuel refuse. Abundancies of raw dung in trash dumps would reflect a decline in its use as fuel and/or fertilizer, marking changes in resource management and agropastoral economics.

Micro-geoarchaeological sediment analyses were used to assess this hypothesis, specifically by tracking spatiotemporal changes in proxies for the disposal of dung, grass cultivar, wood, and fuel resources. A micro-sediment approach was needed to clarify disposal patterns in decomposed resources that are no longer visible to the naked eye. Decomposed organic refuse such as livestock dung and grass cultivars leave behind diagnostic micro-remains that can be extracted from sediments. Microscopic dung spherulites were used as a proxy for the presence of livestock dung, either raw (yet degraded) or as dung fuel ash [62, 63]. Opaline plant phytolith assemblages were used to pinpoint changes in the management of grass and wood resources [64–66]. Changes in wood fuel use were assessed using records of phytoliths and calcitic ash pseudomorphs [67, 68]. Mineralogy *via* Fourier transform infrared spectroscopy (FTIR) was used to distinguish heated, ash rich sediments related to the dumping of domestic hearth refuse [69, 70].

## Materials and methods

**Fig 2:**
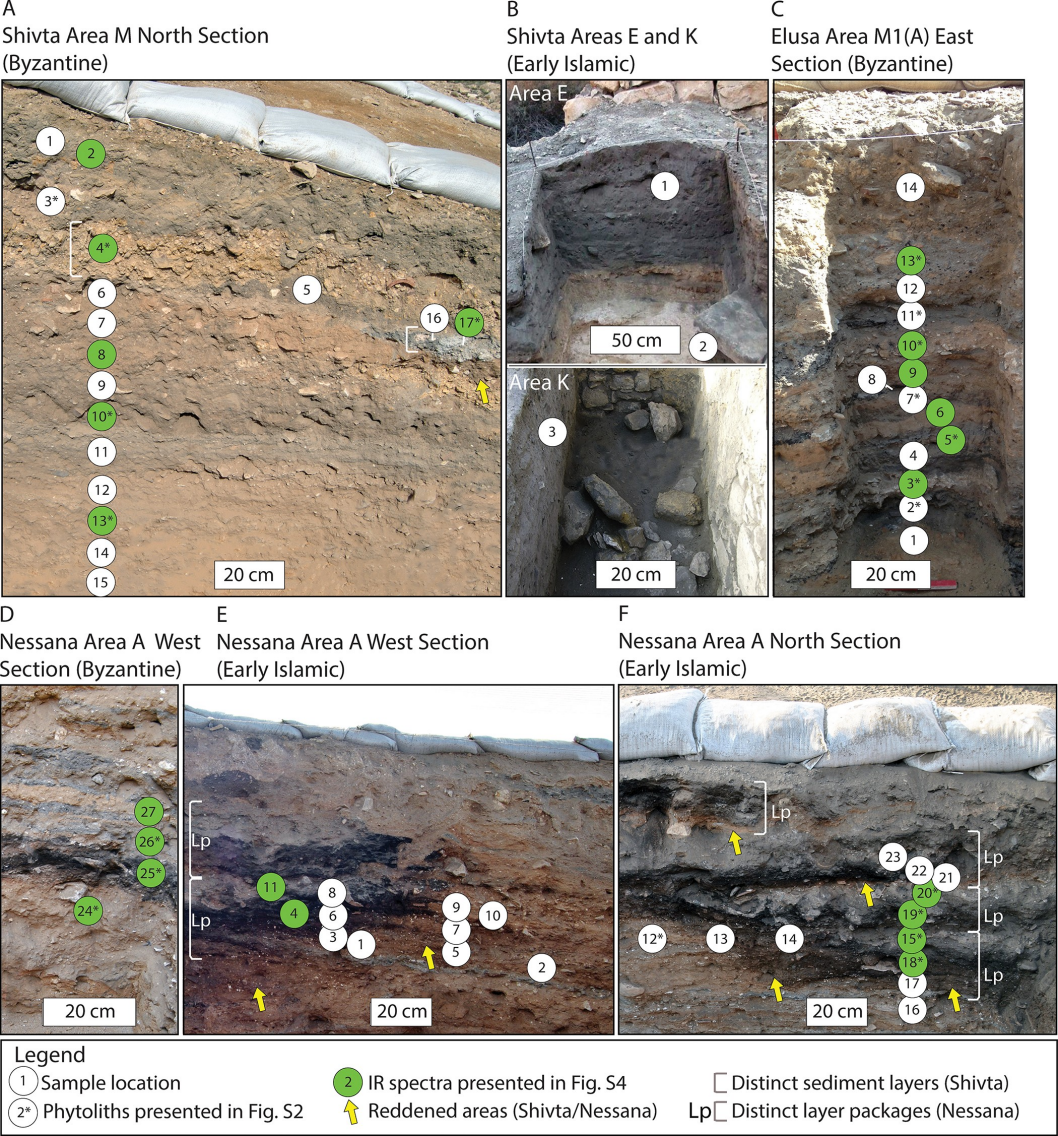
Archaeological trash mound profile sections investigated in this study. (A) The 1.5-meter-thick section excavated into the Area M Byzantine period trash mound at Shivta. The grey lens (samples 16 and 17) and gravel layer (sample 4) are distinct. (B) Early Islamic middens at Shivta Areas E and K. (C) The 1.3-meter-thick section exposed at the Elusa Area A Byzantine period trash mound showing a stratigraphic sequence of alternating light and dark layers, along with two layers comprised primarily of gravels (samples 3 and 10). (D) Sampled Byzantine layers at Nessana. (E) The Early Islamic component of the west section displays a basin-shaped feature comprised of two packages of reddish, black, and grey layers. (F) The Early Islamic component of the north section is also characterized by a similar basin-shaped feature with four packages, each comprised of reddened, blackened, and grey layers.

Stratified sediment layers were distinguished in the field according to their color, texture, structure, consistency, and archaeological contents. Our sampling strategy aimed to capture heterogeneity across each mound’s suite of layers. Point samples (~15–30 g) were collected from exposed profile sections (Fig 2). A 5 × 5 m probe was cut into the Area M trash mound at Shivta. We examined a 1.5 m thick section of the north profile (Fig 2A). Two intramural Early Islamic middens (Areas E and K) were also sampled (Fig 2B). In the hinterland of Elusa, a 2 × 2 m probe was excavated into the Area M1(A) Byzantine trash mound to a depth of 1.3 m (Fig 2C). Two profile sections of the Area A trash mound were studied at Nessana. The lowermost deposits of Late Byzantine origin were sampled from distinct sediment layers along the west profile section (Fig 2D). Four additional bulk samples were collected from Byzantine contexts throughout the mound. Samples from the upper Early Islamic components of the west and north sections were collected from two basin-shaped features and from adjacent sediments (Fig 2E and 2F). We also collected samples from clearly defined intramural features at the Shivta and Nessana villages for comparative purposes (S1 File). Offsite control sediments were collected from typical Negev aeolian-alluvial deposits in the Shivta area. All necessary permits were obtained for the described study, which complied with all relevant regulations. Research was conducted under licenses from the Israel Antiquities Authority (Elusa: G-69/2014, G-10/2015, G-6/2017; Shivta: G-87/2015, G-4/2016; Nessana: G-4/2017).

We determined micro-remain concentrations (plant phytoliths, livestock dung spherulites, and wood ash pseudomorphs), organic matter content (OM), mineralogy, and pH for each sample. The fine fractions (< 2 mm) of all samples were ashed in an electric laboratory furnace (Thermolyne F6000, Thermo Scientific) at 550°C for 4 h to standardize absolute micro-remain concentrations and calculate OM by weight loss-on-ignition [71]. Mineralogical and pH analyses were conducted on unashed samples.

Phytolith, dung spherulite, and ash pseudomorph analyses were conducted using rapid extraction and counting protocols [72, 73]. Systematic counting was carried out using a Nikon Eclipse 50i POL petrographic microscope (16 fields-of-view, 4 × 4 grid). All concentrations are reported as millions of micro-remains per gram of ashed sediment (M/g). The measurement error for this approach is around ± 30%. Phytoliths were counted at 200 × magnification under plane-polarized light (ppl). Phytoliths in multicellular structures were counted individually. Morphotypes were classified at 400 × magnification under ppl. A minimum of 200 morphologically consistent phytoliths were counted on each slide [74–78]. Dung spherulite and ash pseudomorph quantifications were carried out at 400 × magnification under cross-polarized light (xpl) and ppl respectively. We used the ratio of wood ash pseudomorphs to dung spherulites compared against phytolith concentrations/morphotypes to distinguish fuel refuse types [63, 73].

Mineral components were identified using an FTIR spectrometer (Thermo Scientific Nicolet iS5; Omnic 9.3 software; potassium bromide disk method). Sediments were examined for the presence of geogenic and pyrogenic calcite, aragonite, opal, anhydrite, gypsum, and carbonated hydroxylapatite [14, 79, 80]. Heat-altered clay minerals were identified using the procedure detailed in Berna et al. [69], while pyrogenic (wood ash) and geogenic (e.g., limestone) calcites were distinguished using the approach described in Regev et al. [70].

The studied sediments were categorized into archaeological deposit types (DTs) based on their macro- and microscopic characteristics. We used a stepwise categorization procedure based on color, texture, micro-remain concentrations, mineral contents, and modes of formation. Samples were first grouped according to their color and texture. Groups were subdivided according to their dung spherulite concentrations, then ash pseudomorph concentrations, and lastly phytolith concentrations/morphotypes. Concentrations were considered low, medium, or high relative to the archaeological dataset mean. Next, mineralogical data was used to distinguish layers displaying signatures akin to local natural sediments (e.g., loess, limestone, marl) from those with signs of burning (e.g., heated clays, pyrogenic calcite, and aragonite) [70]. Burned sediments were grouped based on the presence or absence of macroscopic stratigraphic evidence for burning directly at the dump site (i.e., stratigraphic layer packages comprised of reddened, blackened, and ash rich sediments) [81]. These were further subdivided according to the dominant fuel source represented [63, 73]. Additional methodological details are presented in S1 File.

## Results and discussion

Trash mounds at Shivta, Elusa, and Nessana revealed layered sediment deposits useful for studying spatiotemporal covariance among resource management, refuse disposal, and agropastoral niche dynamics (Fig 2). Based on pottery typologies, the studied deposits correspond to the Byzantine and Early Islamic periods [11, 22, 23]. The Shivta trash mound dates exclusively to the Byzantine period. Early Islamic trash accumulations located within two abandoned intramural structures were also investigated. A Byzantine trash mound was examined outside Elusa. At Nessana we studied a hinterland mound comprised of Late Byzantine deposits in the lower part of the excavation and Early Islamic deposits toward the top.

**Fig 3:**
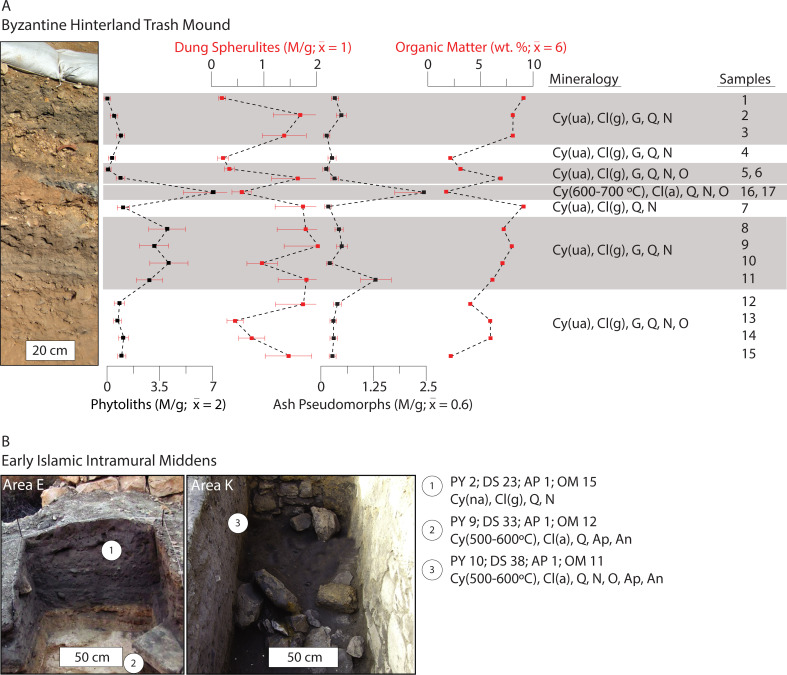
Results of the micro-geoarchaeological analyses conducted on sediments from the Shivta middens. (A) Profile section of the Byzantine hinterland trash mound. Phytolith, dung spherulite, and ash pseudomorph concentrations (M/g), OM content (wt. %), and mineralogy are plotted stratigraphically. Mineralogy is shown for key units. Darker layers are highlighted in grey. The 30% measurement error expected for micro-remains quantification is demarcated with red error bars. (B) Early Islamic middens. Note the lack of clear layering. Heated clays, ash derived minerals, and large amounts of dung spherulites characterize these sediments. Abbreviations: M/g = millions of micro-remains per gram of sediment; wt. % = weight percent; Cy (ua/500°C) = clay (unaltered/heat-altered at the designated temperature); Cl (g/a) = calcite (geogenic/ash); Q = quartz; G = gypsum; N = sodium nitrate; O = opal; Ap = apatite; Ar = aragonite; An = anhydrite.

**Fig 4:**
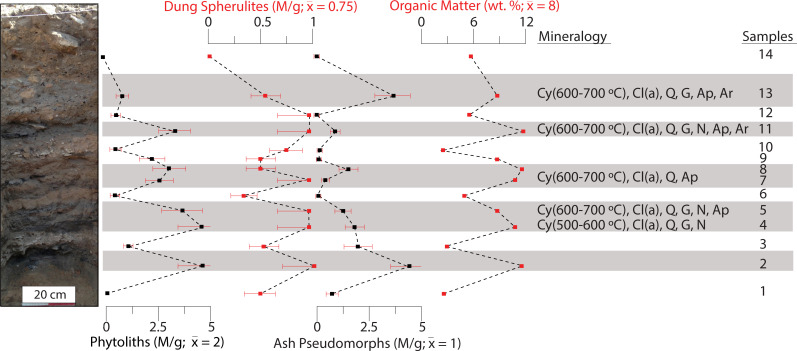
Results of the geoarchaeological analyses conducted on sediments from the Elusa trash mound. Concentrations of phytoliths, dung spherulites, and ash pseudomorphs (M/g), OM (wt. %), and mineralogy are plotted stratigraphically. Darker layers are highlighted in grey. Error bars and abbreviations as in Fig 3. Note the alternating pattern of phytolith, dung spherulite, and organic matter contents corresponding with light and dark layers. Dark layers also commonly display signatures for heat altered clay and ash derived minerals. Mineralogy is shown for these layers.

**Fig 5:**
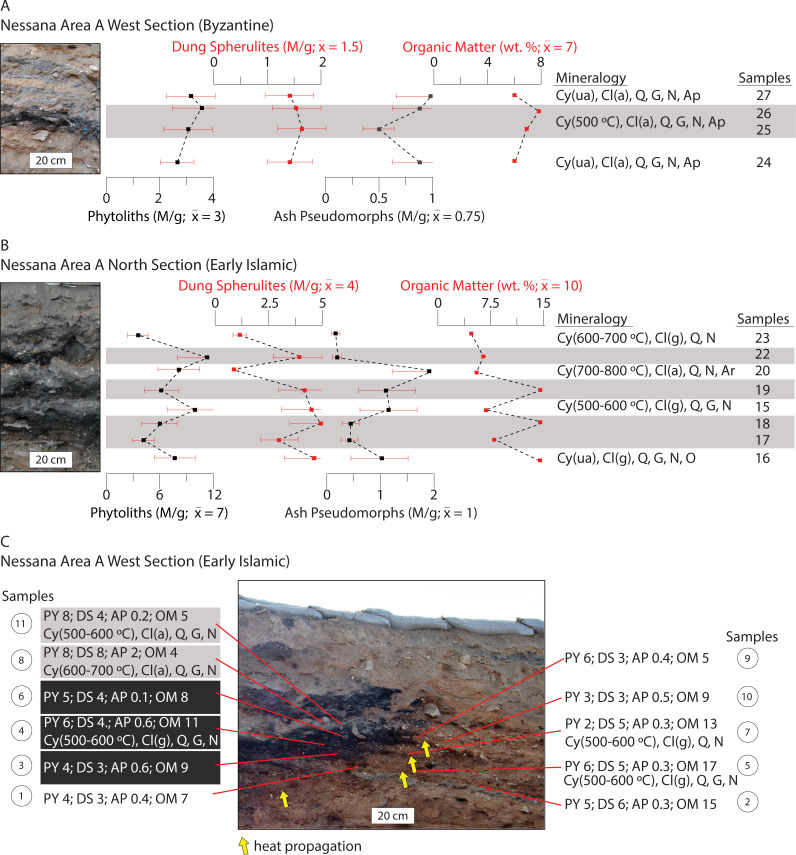
Results of the micro-geoarchaeological analyses conducted on sediments from the Nessana trash mound. Concentrations of phytoliths, dung spherulites, and ash pseudomorphs (M/g), OM (wt. %), and mineralogy are plotted stratigraphically. Darker layers are highlighted in grey. Error bars and abbreviations as in Fig 3. (A) The Byzantine layers have internally consistent micro-remain characteristics. (B, C) The grey layers in these Early Islamic contexts display lower organic matter than the darker layers and mineralogical signatures for heated clay. Dung spherulite concentrations are relatively high throughout the tested sediments. Representative mineralogy is shown.

**Fig 6:**
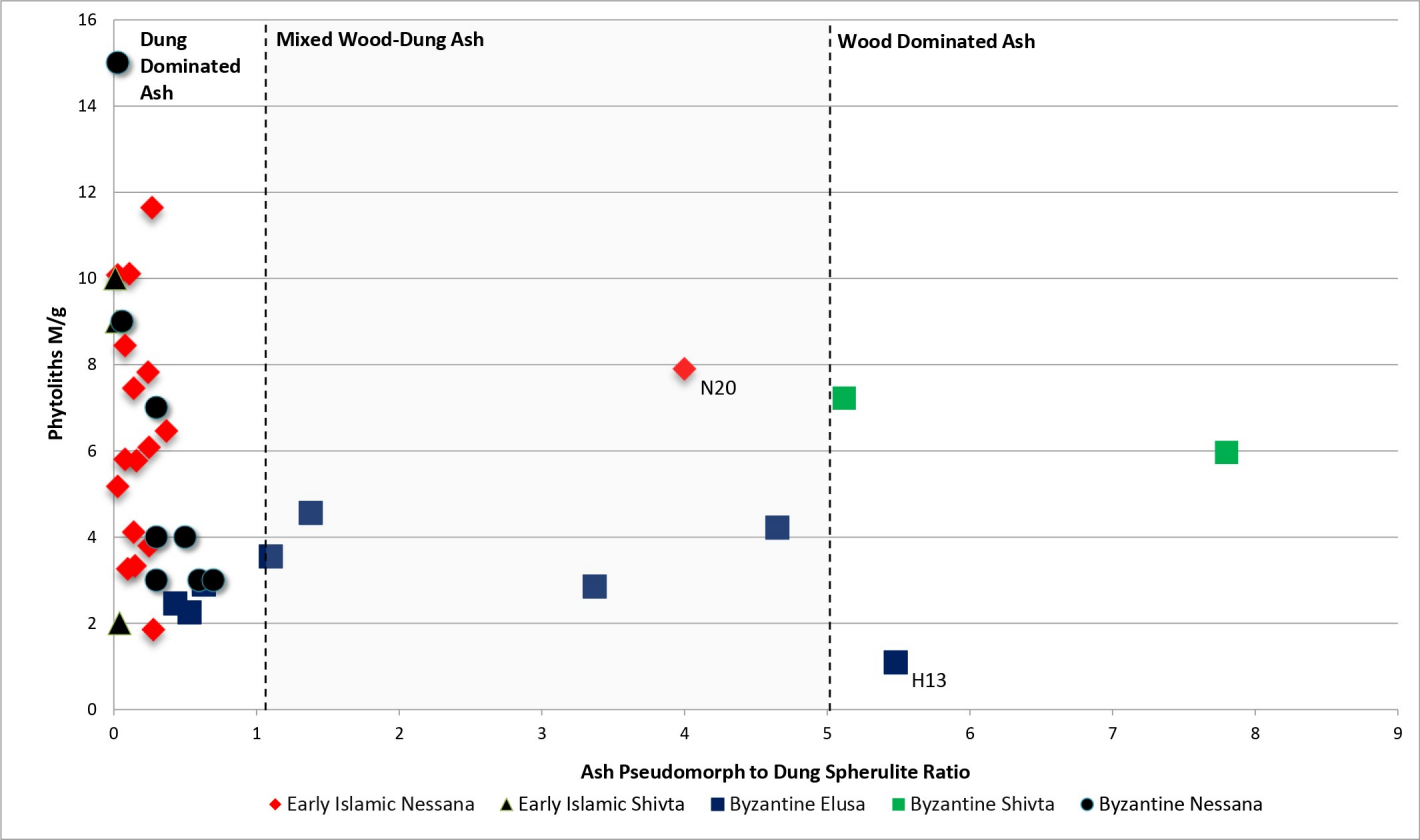
Ash pseudomorph to dung spherulite ratios plotted against phytolith concentrations for trash layers containing burned refuse. Fuel sources are inferred using this plot (after Gur-Arieh et al. 2013, 2014). The graph is sectioned into regions of dung dominated ash (lower PSRs and higher phytolith concentrations), wood dominated ash (higher PSRs and lower phytolith concentrations), and mixed dung/wood ash (PSR ranging between 1 and 5). Dung fuel refuse is present at all sites. The uppermost dark layer from Elusa (H13) and the grey lens from Shivta contain wood dominated ash. One sample from early Islamic Nessana (N20) is an outlier for the site, which likely relates to dung spherulite decomposition during high temperature burning.

**Fig 7:**
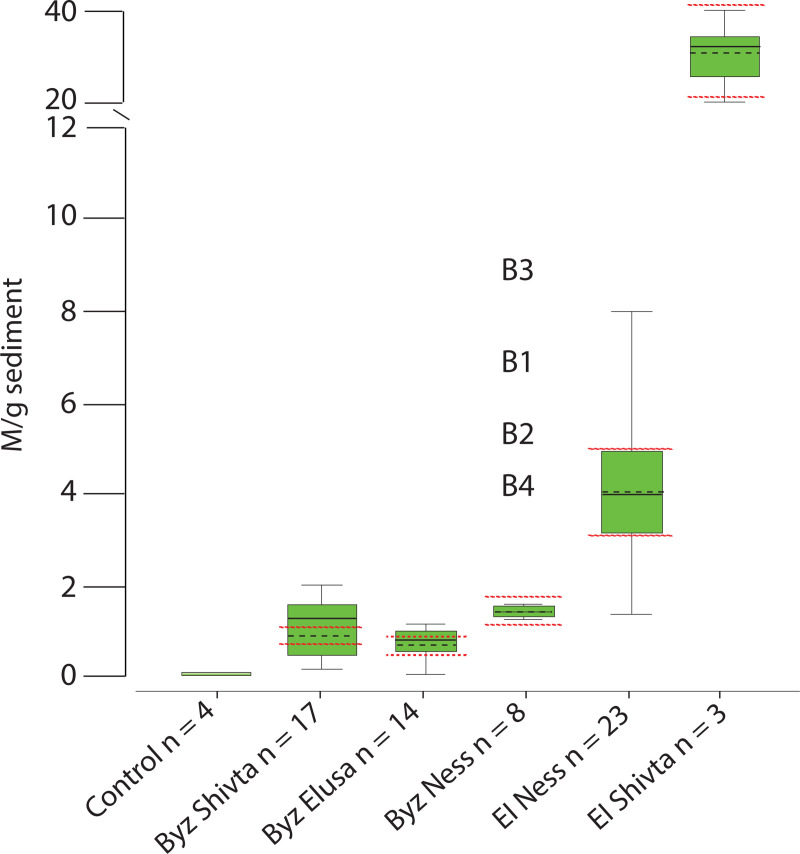
Boxplot comparison of dung spherulite concentrations across the Shivta, Elusa, and Nessana sediments. The box represents the interquartile range, and the upper and lower whiskers represent the maximum and minimum values respectively. The internal solid line is the median, while the dashed line is the mean. Dashed red lines represent the mean 30% error typical of micro-remains quantification analyses. Outliers are listed by sample number (i.e., bulk sediments from Byzantine Nessana).

Offsite control sediment samples contained negligible to no micro-remains (< 0.07 M/g) and OM contents of ~ 7%. Their mineralogical signatures were typical of local natural sediments (e.g., quartz, clay, geogenic calcite) [cf. 61] (S3 Table in S1 File). Generally, trash mound sediments were comprised of brown, yellowish, reddish, black, and grey soft silty loams, with or without gravel, forming layers and lenses of variable lateral extent, thickness, and directionality. Micro-remain concentrations in the trash deposits were considered low, medium, or high relative to the archaeological dataset mean. Micro-remain means for the archaeological dataset are as follows: phytoliths = 4 M/g; dung spherulites = 4 M/g; ash pseudomorphs = 1 M/g. Results are presented in Figs 3–7 and S1 File (S1-S6 Figs in S1 File and S1-S3 Tables in S1 File).

### Byzantine Shivta

Most of the brown silt loam deposits from the mound contained low phytolith and dung spherulite concentrations of ~ 1 M/g or less, OM contents of ~ 6%, and no mineralogical evidence for heat-altered clays or ash (Fig 3A samples 1–3 and 12–15, and S4A Fig in S1 File). Both grass and wood phytolith morphotypes were identified (e.g., elongate dendritics and irregulars respectively) (S2 Fig in S1 File). A distinct light brown-yellowish gravel rich deposit near the top of the profile displayed very low micro-remain concentrations and OM contents (< 0.5 M/g and 2% respectively) (Fig 3A, sample 4). Mineralogical characterization suggested the gravels are marl, a calcite rich mudstone common in the bedrock of the Shivta area (S4A Fig in S1 File) [82]. A reddish area capped with a grey lens was found below the gravel deposit (Fig 3A, samples 16 and 17). The lens contained high phytolith concentrations (~ 7 M/g). Phytoliths were primarily from woody plants (e.g., morphotypes including discoids and irregulars) (S2 Fig in S1 File). Ash pseudomorph concentrations were high relative to the dataset mean (~ 3 M/g), while dung spherulite concentrations and OM contents were low (0.5 M/g and 2% respectively). Pyrogenic calcite indicative of wood ash and clays exposed to temperatures between 600°C to 700°C were identified (S4A Fig in S1 File).

### Byzantine Elusa

An alternating sequence of light brown and darker grey deposits was documented in the studied trash mound (Fig 4). Fine textured, lighter sediment deposits (samples 1, 6, 9, 12, and 14) had low micro-remain concentrations (< 1 M/g) and OM contents (~ 5%). Mineralogical signatures were typical of local natural sediments (e.g., quartz, clay, geogenic calcite). Dung spherulite concentrations were very low, averaging 0.4 M/g. Two lighter deposits contained angular limestone flakes and sub-angular fragments of unheated clay (S4B Fig samples 3 and 10 in S1 File). Darker deposits contained higher micro-remain concentrations than the lighter deposits (Fig 4, samples 2, 4, 5, 7, 8, 11, and 13). On average, phytolith concentrations were five times higher, while dung spherulite, wood ash pseudomorph, and OM contents were double those of the lighter layers. Mineralogical signals for ash (pyrogenic calcite, carbonated hydroxylapatite, aragonite) and clays heated to temperatures between 600°C and 700°C were common (S4B Fig in S1 File). Phytolith morphotypes representing grasses and woody plants were present in varying proportions (e.g., elongate dendritics and irregulars respectively) (S2 Fig in S1 File).

### Late Byzantine Nessana

The brown, grey, and black sediment deposits comprising the Late Byzantine component of the Nessana mound had phytolith concentrations reaching 4 M/g (Fig 5A). Grass morphotypes (e.g., elongate dendritics and echinates [elongate dentates]) from grass inflorescence were well represented (S2 Fig in S1 File). Dung spherulite concentrations were low in the tested layers (~ 1.5 M/g). Ash pseudomorph concentrations were average (1 M/g), except for a low concentration of 0.5 M/g in sample 25. The clays present in samples 25 and 26 were heated at temperatures around 500°C (S4C Fig in S1 File). Dark brown bulk sediment samples contained high phytolith and dung spherulite contents ($\bar{x}$ = 9 M/g and $\bar{x}$ = 6 M/g respectively). All but one of the samples contained mineralogical evidence for ash in the form of pyrogenic calcite (S4C Fig in S1 File).

### Early Islamic Shivta

The dark grey sediments comprising the midden features investigated at Areas E and K had no clear layering (Fig 3B). Phytolith concentrations were as high as 10 M/g. Dung spherulite concentrations were extremely high, ranging from 23–38 M/g. Sediments from both areas contained clays heated at temperatures between 500°C and 600°C, along with pyrogenic calcite (ash), carbonated hydroxylapatite, and anhydrite.

### Early Islamic Nessana

The loose, dark brown sediments found throughout much of the upper part of the profile had dung spherulite concentrations as high as 6 M/g. Phytolith concentrations surpassed 7 M/g (Fig 5B and 5C, samples 1, 2, 5, 7, 9, 10, 12, 13, and 16). Phytolith assemblages also showed a tendency toward domesticated grass morphotypes (e.g., elongate dendritics and echinates [elongate dentates] from inflorescence) (S2 Fig in S1 File and S2 Table in S1 File). There were no signs of heated clays nor mineralogical signatures for pyrogenic calcite (ash) in most of these locations.

Sediments from two distinct basin-shaped features were characterized (Figs 2E, 2F and 5B and 5C). The basal component of the west profile feature was distinguished by a large reddened area that expanded over existing sediment deposits (~ 0.5 m^2^). The black area above this propagated laterally through extant layers. A grey deposit was documented above the black area. A similar sequence repeated above this (Fig 5C). The north section was comprised of four such sequences (Fig 5B). The black areas (samples 3, 4, 6, 14, 17, 18, 19, and 22) had OM contents of ~10–15%. Only samples 5 and 7 contained signals for clays heated at 500°C to 600°C. Clays in the grey layers (samples 8, 11, 15, 20, and 23) were exposed to temperatures between 500°C and 800°C. OM contents were low (< 5%) compared to the black areas. Pyrogenic calcite (ash) was identified in samples 8, 11, and 20. Clays heated at temperatures as high as 700°C—800°C and aragonite were identified in sample 20 (S4D Fig in S1 File).

### Micro-geoarchaeological investigations detected the disposal of different refuse types

Owing to neutral/slightly basic pH levels and dry conditions, the studied micro-geoarchaeological proxies were well preserved, displaying only minor degradation of micro-remains and excellent preservation of inorganic components [14]. It is likely, however, that calcitic dung spherulites were thermally decomposed and therefore underrepresented in sediments exposed to temperatures surpassing 600°C [63, 73, 83].

We distinguished seven refuse deposit types (DTs) using the findings detailed above (summarized in Table 1). Based on less than average micro-remain and OM contents, and mineral signatures typical of local natural sediments, DT-1 is interpreted to represent the deposition of small amounts of organic wastes that have likely been diluted with loess (aeolian sediment). DT-1 was the dominant sediment type recorded at the Shivta mound (Fig 3A). It was also identified at Elusa (Fig 4). Notably, raw livestock dung was poorly represented in these locations.

**Table 1:** Deposit Types (DT) defined according to distinct macro and microscopic sediment characteristics and formation processes.

Deposit Type	Macroscopic characteristics	Dung spherulite concentration	Ash pseudomorph concentration	Phytolith concentration	Phytolith morphotypes	Organic matter	Other identified components	Site, Period: Corresponding Samples	Interpretation
**DT-1**	soft, light or dark brown loam	1 M/g ± 0.7	0.2 M/g ± 0.1	1 M/g ± 1	monocot: 44 ± 30%	7 ± 2%	no heated clay or ash related minerals	Shivta, Byzantine: 1, 2, 3, 5, 6, 7, 8, 9, 10, 11, 12, 13, 14, 15	Small amounts of dumped organics diluted with loess
					dicot: 41 ± 20%			Elusa, Byzantine: 1, 6, 9, 12, 14	
**DT-2**	soft, light brown or yellow loam with gravel	0.5 M/g ± 0.2	1 M/g ± 2	1 M/g ± 0.5	monocot: 23 ± 16%	3 ± 1%	no heated clay or ash related minerals; gravels include limestone, marl, mud	Shivta, Byzantine: 4	Dumped masonry/ mud construction debris
								Elusa, Byzantine: 3, 10	
					dicot: 62 ± 23%				
**DT-3**	lens (40 cm wide; 20 cm thick); soft, grey loam overlying a reddened base	0.5 M/g ± 0.02	3 M/g ± 1	7 M/g ± 1	monocot: 21%	2 ± 1%	heated clay + ash related mineral (calcite)	Shivta, Byzantine: 16, 17	*In situ* wood fueled fire
					dicot: 76%				
**DT-4**	soft, grey, black loam	1 M/g ± 0.3	2 M/g ± 1	3 M/g ± 1	monocot: 45 ± 18%	11 ± 1%	heated clay + ash related minerals (calcite, apatite, aragonite)	Elusa, Byzantine: 2, 4, 5, 7, 8, 11, 13	Dumped mixtures of combusted dung and wood fuel refuse
					dicot: 40 ± 19%				
**DT-5**	soft, brown, black, grey loam	11 M/g ± 13	1 M/g ± 1	6 M/g ± 4	monocot: 39 ± 13%	12 ± 4%	heated clays + ash related minerals (calcite, apatite, aragonite, anhydrite)	Nessana, Byzantine: 24, 25, 26, 27, bulk 1, bulk 2, bulk 3, bulk 4	Large amounts of combusted then dumped dung fuel refuse
					dicot: 32 ± 8%				
								Shivta, Early Islamic: 1, 2, 3	
**DT-6**	soft, dark brown loam	5 M/g ± 1	0.5 M/g ± 0.3	4 M/g ± 2	monocot: 50%	12 ± 4%	some signs of heated clays; no ash related minerals	Nessana, Early Islamic: 1, 2, 5, 7, 9, 10, 12, 13, 16	Dumped raw dung refuse
					dicot: 19%				
**DT-7**	basin shaped (2–3 m wide; ~1 m thick); internal organization of layers: red (bottom), black (middle), grey (top)	4 M/g ± 1	1 M/g ± 0.5	7 M/g ± 2	monocot: 53 ± 9%	7 ± 3%	heated clays + ash related minerals (calcite, aragonite)	Nessana, Early Islamic: 3, 4, 6, 8, 11, 14, 15, 17, 18, 19, 20, 21, 22, 23	Dung ash produced by *in situ* burning atop the trash mound
					dicot: 21 ± 5%				
**Offsite control**	light brown, yellow silty loam	0.07 M/g ± 0.03	0	0	0	7 ± 3%	geogenic calcite, quartz, clay, gypsum, sodium nitrate	Shivta Area, Recent: 1, 2, 3, 4	Aeolian/alluvial sediments

Means and standard deviations for dung spherulites, ash pseudomorphs, phytoliths (concentrations and morphotypes; monocots are non-woody plants, dicots are woody plants), and organic matter are presented. Ash related minerals documented include calcite, apatite, aragonite, and anhydrite.

DT-2 contained limestone gravel, mud and marl fragments, and light-colored loam with characteristics typical of local natural sediments. Found in the Byzantine mounds outside Shivta and Elusa, these deposits are likely the remains of dumped masonry and mud-based construction debris (Figs 3A and 4).

DT-3, a lens displaying evidence typical of a small fire at the dump site (i.e., *in situ*), was found at the Byzantine mound outside Shivta (Fig 3A). *In situ* fires typically have distinct basin- or lenticular-shaped layer packages formed by fuel consumption and heat propagation, specifically a reddened (rubified) layer capped with either a grey/white ash rich deposit (high temperature combustion), a carbonized/blackened layer (lower temperature combustion), or both [81, 84]. An abundance of phytoliths from woody plants and wood ash pseudomorphs, as well as low dung spherulite concentrations, indicated that wood was the dominant fuel resource used in this location (Fig 6 and S2 Fig in S1 File).

DT-4 is comprised of dark grey sediments found in the Byzantine dump outside Elusa (Fig 4). Based on phytolith morphotypes and the ratios of wood ash pseudomorphs to dung spherulites, these deposits represent varying amounts of combusted livestock dung and wood fuel refuse (Fig 6 and S2 Fig in S1 File) [63]. The absence of stratigraphic evidence for *in situ* fire confirmed that these spent fuels were dumped at the mound. Fuel wastes were reasonably cleaned from domestic hearths and dumped at the trash mound.

DT-5 represents the dumping of large amounts of combusted dung fuel refuse. These deposits were identified in the Byzantine and Early Islamic trash disposal contexts at Nessana and Shivta respectively (Figs 3B and 5A). They were characterized by large amounts of dung spherulites associated with heated clays (Fig 6 and S4 Fig in S1 File). There was no stratigraphic evidence for *in situ* burning in either context. The thick and uniform intramural midden deposits investigated at Early Islamic Shivta had no clear layering, which suggests the consistent dumping of combusted dung fuels (Fig 3B). Like DT-4, this refuse was likely produced in domestic hearths, raked out, and later dumped at the trash mounds.

DT-6 uniquely represents the dumping of raw, unburned herbivore dung. As the mound is unequivocally a trash dump, including sharp refuse (e.g., broken pottery, glass, bone, metal, etc.), dung deposition through animal penning or grazing is unlikely. The deposits were identified exclusively in the Early Islamic component of the trash mound outside Nessana (e.g., Fig 2E, toward the right). Dung spherulite concentrations were roughly five times higher than DT-1. There were no stratigraphic, micro-remain, or mineralogical indications of burning.

Identified exclusively in the Early Islamic component of the Nessana dump, DT-7 represents the periodic burning of livestock dung directly atop the mound (i.e., the burning of DT-6) (Fig 2E and 2F). These basin-shaped packages of superimposed reddened, blackened, and grey ash rich layers were formed through several *in situ* burning events. Clays from the grey layers were exposed to temperatures between 500°C and 800°C, suggesting these locations were the core areas of fuel combustion (Fig 5B and 5C and S4D Fig in S1 File). Dung concentrations may have originally been higher in these locations. Burning likely reduced spherulite concentrations [73, 83]. Heat was transferred from these central combustion locations through adjacent sediments, which was indicated by reddening (rubification) and carbonization overprinting extant stratigraphy (Fig 2E and 2F). Blackened areas (some containing darkened, heat altered dung spherulites) represent carbonization caused by heating in oxygen deprived atmospheres at temperatures between roughly 300°C and 500°C (S1C and S3C Figs in S1 File) [81, 83, 84]. The higher OM contents (~ 10–15%) relative to grey layers (~ 4–6%), lack of mineralogical evidence for ash, and a general absence of heated clays confirmed that most of these layers were not heated to temperatures surpassing 500°C. However, samples 5 and 7 did contain clays heated at 500°C to 600°C, indicating stronger heat propagation into these locations (Fig 5C).

High concentrations of dung spherulites, low wood ash pseudomorphs, and low ratios of wood to grass phytoliths throughout the layer packages highlighted livestock dung as the dominant fuel source (Fig 6 and S2 Fig in S1 File). Together, the dung spherulite concentrations, multiple stratigraphic packages indicative of *in situ* burning, and the overall size of these features (~ 2–3 m wide; ~1 m thick) suggests that significant amounts of dung were managed at the dump site through several burning episodes (Fig 5B and 5C). Owing to compression over time, the deposits were likely much thicker originally. The burned areas appear too large to have been simple campfires.

### Trash deposits revealed sustainability and community-scale change in resource management

The deposit types characterized at the trash disposal sites provide new evidence for sustainability and change in resource management. The most conspicuous shift we detected relates to the management of livestock dung (Fig 7). Community-scale changes in dung resource use and disposal are represented in trash deposit sediments by varying concentrations of calcitic dung spherulites and wood ash pseudomorphs, along with mineralogical and stratigraphic evidence for burning.

The use of livestock dung as a sustainable fuel resource was confirmed by the disposal of dung ash at Byzantine Elusa and Nessana, as well as Early Islamic Shivta and Nessana (DT-4 and DT-5). The widespread use of dung fuels suggests that domestic heating needs placed little pressure on hinterland vegetation during Late Antiquity.

Low dung spherulite concentrations in the unburned sediments at Byzantine Shivta and Elusa indicate that raw dung comprised only a small fraction of the wastes dumped at the study locations (DT-1) (Fig 7). It is doubtful that this represents diminutive sheep/goat livestock economies, considering the overrepresentation of ovicaprine remains recovered from the sites (80% of the assemblage at Shivta and 88% at Elusa) [11, 22]. Coupling the domestic and commercial importance of large-scale agriculture with farmers’ concerns for improving and maintaining arable land, we propose that livestock dung offered a widely accessible and sustainable fertilizer resource. Instead of being disposed of in trash dumps, dung would have been spread in agricultural plots. If correct, our proposal implies stability in the functioning of the agropastoral socio-ecological niche during the Byzantine period. Additional research is required to improve our ability to directly identify the use of herbivore dung fertilizers in field, orchard, vineyard, and other agricultural contexts.

In sharp contrast to the sustainable use of dung for fuel, and reasonably for fertilizer as well, raw dung was dumped and burned atop the mound outside Early Islamic Nessana (DT-6 and DT-7 respectively). This is the first evidence of its kind from the Negev confirming the management of dung *via* controlled incineration. It is unlikely that this disposal strategy relates to increased dung production from growing herd sizes. While recent zooarchaeological findings do confirm the presence of sheep/goat livestock in the Nessana area, there is no evidence indicating that herds were exceptionally large. In fact, fewer sheep/goat remains were recorded in Early Islamic trash deposits compared to the Late Byzantine period [22]. The growth of pastoral sedentism throughout the Early Islamic period is an alternative explanation. Several studies present evidence for the ruralisation of the urban heartland: populations and infrastructure were fading, and herders from the south were settling into the area, operating more intensively in rangelands and farmlands connected to declining villages [44, 85–91]. This would have increased dung production in and around Nessana. Some of this surplus may have been used for fertilizer, some for fuel, and the excess was dumped and incinerated during regular village maintenance. Our evidence for the latter indicates that a considerable amount of this resource was not used as fertilizer, nor as fuel for that matter.

We propose that this dung management strategy represents a decline in large-scale local agriculture superimposed atop the already gradually eroding regional market system. At Nessana, the disposal of potentially valuable fertilizer implies there was little incentive to ensure the profitability of agriculture beyond domestic needs, which we see as a community-scale response to disruption and restructuring within the commercial agriculture sector paired with the expansion of village-tethered herding in the heartland [20, 26, 44, 86, 89]. Recent research is now indicating that agropastoral practices were likely not heavily influenced by climate change or pronounced environmental degradation [20, 37]. Another type of disruption is recorded in the Nessana papyri (512–689 CE). Several of the Arabic documents written after the fall of Byzantine hegemony speak of the difficulties Nessana residents had in paying rising taxes, particularly those levied against farmlands and produce [85, 90]. Christian pilgrimage through Nessana also came to a halt, and trade networks in the region were crumbling [19, 20, 92–95]. These circumstances reasonably contributed to reducing the value and/or need for commercial produce. Moreover, the center of the Islamic state moved from the Levant to Mesopotamia by the 9^th^ century CE [19]. This led to a major downturn in governmental contributions to the management of the extensive labor and runoff harvesting systems needed to ensure the profitability of large-scale market-oriented agriculture [20, 86]. Under these economic and political circumstances, or *conjoncture* as Braudel [3] put it, Nessana appears to have been transforming from an agricultural center into a more rural community persisting from smaller-scale domestic farming, semi-sedentary herding, and wild game hunting [20, 22, 45, 85–91; see 22 for evidence of hunting].

## Summary and conclusion

Ancient hinterland trash mound features are important sources of evidence for community-scale resource management, economics, and socio-ecological dynamics. We found that dung was used as a sustainable fuel resource during both the Byzantine and Early Islamic periods. Conversely, significant amounts of raw dung were dumped and then managed by incineration outside Early Islamic Nessana. These results provide support for the hypothesis that agropastoral dynamics are reflected in the management of livestock dung. They highlight a previously unrecognized community-scale response to disruption within the long-standing agropastoral socio-ecological niche. The trash mound archive outside Early Islamic (Umayyad) Nessana specifically revealed a community-based response to top-down governmental pressures. We propose that strategies aimed toward securing dung surpluses for prospective use as fertilizers were downsized in parallel with the decline of the commercial agriculture sector. Smaller-scale domestic agriculture and village-tethered herding appear to have been central to Early Islamic economics at Nessana. Our findings are consistent with archaeological and historical evidence that connects major economic and socio-ecological changes (i.e., the collapsing agricultural niche) to taxation, a shift toward semi-sedentary herding in the former village heartland, ruralisation, and diminishing governmental support during the period leading up to the turn of the first millennium CE. They give additional weight to the socio-economic factors involved with the transformation of community-centered agropastoral niches in the Negev of Late Antiquity.

Along with this, the study demonstrates the importance of investigating open-air spaces beyond the confines of settlement walls when assessing the causes and consequences of changing resource management strategies. Micro-sedimentary investigations are particularly important in classical urban studies. The approach contributes to resolving stationarity and change in the use of organic resources, including grass cultivars, wood, and livestock dung, that have decomposed and are no longer visible to the naked eye. Broadly, the study provides comparative data, and it demonstrates the high potential of archaeological trash proxies in studies aiming to detail and explain wide-ranging diversity in the processes conditioning socio-ecological transformations, as well as how communities contribute and respond to such transformations.

## Supporting information

S1 File Appendix. (PDF)

S2 File (PDF)
