# Supplementary material for: Byzantine—Early Islamic resource management detected through micro-geoarchaeological investigations of trash mounds (Negev, Israel)
Source: PLoS One. 2020 Oct 14;15(10):e0239227. doi: 10.1371/journal.pone.0239227 (PMC7556535; doi:10.1371/journal.pone.0239227)
Supplement: S1 File — (PDF) [file pone.0239227.s001.pdf]

S1 File. Appendix

Byzantine - Early Islamic resource management detected through micro-geoarchaeological investigations of trash mounds (Negev, Israel)

Don H. Butler[1*, #a], Zachary C. Dunseth[1, 2, #b], Yotam Tepper[3, 4], Tali Erickson-Gini[5],
Guy Bar-Oz[3*], and Ruth Shahack-Gross[1*]

1 Laboratory for Sedimentary Archaeology, Department of Maritime Civilizations, Recanati Institute of Maritime Studies, Leon H. Charney School of Marine Sciences, University of Haifa, Haifa 3498838, Israel

2 Jacob M. Alkow Department of Archaeology and Ancient Near Eastern Cultures,
Tel Aviv University, Tel Aviv 6997801, Israel

3 Zinman Institute of Archaeology, University of Haifa, Haifa 3498838, Israel

4 Israel Antiquities Authority, 61012, Tel Aviv, Israel

5 Israel Antiquities Authority, Archaeological Division, P.O. Box 271, Omer 8496902, Israel

* Corresponding Authors: D.H. Butler (dhbutler3@alaska.edu); G. Bar-Oz (guybar@research.haifa.ac.il);
R. Shahack-Gross (rgross@univ.haifa.ac.il)

#a Current Address: Department of Anthropology, University of Alaska Fairbanks, P.O. Box 757720, Fairbanks, Alaska 99775-7720, USA

#b Current Address: Joukowsky Institute for Archaeology and the Ancient World, Brown University, Providence, Rhode Island 02906, USA

Additional site details

Shivta was established during the Early Roman period (c. late 1st - 2nd century CE) and reached its economic peak under Byzantine rule (5th - 6th centuries CE) (Fig 1B) [24]. This peak was characterized by extensive and highly maintained runoff agriculture infrastructure, the expansion fruit orchards and grape/wine and olive production, the establishment of public facilities, and an influx of exotic trade goods [24, 45, 53]. Shivta's population began to decline during the 7th century CE, and it was abandoned by the end of the 9th century CE [19, 24]. Many heartland settlements were in decline by the end of Byzantine hegemony, and they continued to erode under Early Islamic administration between the 7th and 10th centuries CE [20, 86, 87].

Elusa began as way station on the Nabatean/Roman Incense Road (or Petra-Gaza Road) as early as the 3rd century BCE [92, 93]. By the 4th century CE, it had grown into a true city (*polis*) that served as the capital of local Byzantine administration (Fig 1C) [11]. Many public and industrial facilities were built, including baths, churches, and pottery workshops. Economics and staple resources were akin to those supporting Shivta. Christian pilgrimage through the city was also an important source of income [91]. Recent radiocarbon dating of materials from hinterland trash mounds pinpoints the cessation of trash disposal to the Late Byzantine period (mid-6th century), suggesting a serious decline in the functioning of the city [11]. Parts of the former urban space were used during the later Umayyad period [94].

Nessana was established as early as the 3rd century BCE as a station along a secondary Nabataean trade route (Fig 1D). The village prospered with the establishment of Byzantine hegemony apparently because of increased revenue from the production and export of wine, as well as Christian pilgrimage [19, 91]. The Nessana papyri, written between 512 and 689 CE, provide numerous details on socioeconomics during the Byzantine and incipient Early Islamic periods, including taxation and produce types (e.g., wheat, barley, legumes, figs, grapes, and olives). The settlement appears to have been largely abandoned by the mid-9th century CE [19, 90].

Additional methodological details and results

A. Phytoliths

Preliminary microscopic scanning of the sediment samples was done to gain a general understanding of the micro-remains present. Sediment fine fractions were mounted in Entellan (Merck) and explored using a Nikon Eclipse 50i POL petrographic microscope at 200 × and 400 × magnification under both plane-polarized (ppl) and cross-polarized (xpl) light. The visibility of the micro-remains was obfuscated in samples from layers containing abundant charred remains and organic matter. All samples were ashed in order to increase the clarity of the micro-remains.

The extraction procedure mobilizes micro-remains into the supernatant of a sodium polytungstate medium (SPT; $Na_6[H_2W_{12}O_{40}]$; specific gravity = 2.4 g/ml). For phytolith extraction, sediments were sieved through 150 µm mesh. Between 20 and 30 mg of sieved sediment was weighed on an analytical balance (Sartorius, Entris) and placed in a 2 ml Eppendorf centrifuge tube. Carbonates were dissolved by adding 50 µl of hydrochloric acid (6 N

HCl) and vortexing (Boeco V-1 Plus). Once effervescence was complete, 450 µl of SPT was added, followed by vortexing, sonication for 10 min (Elma S 15 H), and lastly centrifugation for 5 min at 5000 rpm (Eppendorf 5418). The supernatant was extracted *via* pipette, placed in a new 0.5 µl centrifuge tube, and vortexed. A 50 µl aliquot of the supernatant was slide mounted under a 24 × 24 mm cover slip. Counting and calculation procedures are described in the main text.

Phytolith morphotypes common in the study region listed in S1 Table. Short cell (e.g., bilobates, saddles, and rondels) and long cell (e.g., psilates, dendriforms, and echinates [dentates]) morphologies were used to identify grasses. Dendriform percentages clarified the contributions of wild and domestic grasses to the assemblages, while grass leaf/stem to inflorescence ratios were used to estimate the contributions of chaff, straw, and whole grasses [81]. Morphotypes such as irregulars, discoids, and spheroids were used to infer the presence of dicotyledonous (dicot) leaves, wood, and bark [75, 76]. Examples of morphotypes discovered in the study are provided in S1 Fig . Opaline phytoliths with delicate morphologies or low surface area to bulk ratios are expected to undergo partial dissolution in highly alkaline sediments (pH ~10) [14].

S1 Table. Phytolith morphotypes typical of the study region. * = morphotypes not discovered in this study.

**Morphotype**	**Assignment**
Epidermal Appendage (Hair Base); Platelet (Epidermal Skeleton); Polyhedral; Tracheid	Dicot Leaves
Discoid Rugulate; Discoid Psilate; Ellipsoid Psilate; Ellipsoid Rugulate; Irregular Psilate; Irregular Rugulate; Spheroid Psilate; Spheroid Rugulate*	Dicot Wood/Bark
Long Cell Dendritic (Elongate Dendritic); Long Cell Echinate (Elongate Dentate); Long Cell Verrucate (Elongate Verrucate); Papillae (Papillate)	Grass Inflorescence
Bulliform Cell Cuneiform (Bulliform Flabellate); Bulliform Cell Parallelipedal (Blocky); Cylindroid Echinate; Cylindroid Psilate; Cylindroid Rugulate; Cylindroid Sinuous*; Cylindroid Psilate Bulbous; Epidermal Appendage (Prickle) (Acute bulbosus); Long Cell Psilate (Elongate Entire); Long Cell Sinouous [Elongate Sinuate]; Long Cell Wavy [Elongate Sinuate]	Grass Leaves/Stems
Short Cell Bilobate; Short Cell Polylobate*; Short Cell Saddle; Short Cell Rondel; Short Cell Rondel Tower; Short Cell Trapeziform (Trapezoid); Crenate; Crenate Long*	Grass Leaves/Stems or Inflorescence
Spheroid Echinate	Palm
Cyperaceae Type (Hat-Shaped)*	Sedges
Parallelpiped Thin Psilate; Parallelepiped Blocky Psilate; Parallelepiped Blocky Rugulate; Stomata; Fibre; Parallelepiped Elongate Psilate; Epidermal Appendage	Indeterminate

We used the percentage of dendritic phytoliths to estimate the relative proportions of wild and domesticated grasses represented. A percentage greater than seven indicates a dominance of domestic grasses [85, 86]. Grass leaf/stem to grass inflorescence ratios (i.e., cuneiforms, cylindroids, etc. v. dendritics [elongate dendritics], echinates [elongate dentates], etc.) provided further information on weather organs from entire grass plants were represented in sedimentary archives. Ratios ranging from 1.2 to 3.2 reflect the presence of whole grass plants, while those above 3.2 indicate the selection of leaf/stem grass organs over inflorescence (i.e., straw). Ratios ranging between 0.6 and 1.2 suggest a tendency toward more inflorescence, while those below 0.5 suggest the selection of grass inflorescence (i.e., chaff) [79]. The results of these analyses are presented in S2 Table. Grasses can contain upward of 20 times more phytoliths than wood. Dung from grass fed livestock typically contain high phytolith concentrations [15]. Names in parentheses indicate ICPN 2.0 nomenclature [77].

S1 Fig. Examples of phytolith morphotypes identified in this study. 1: rondel; 2: parallelepiped psilate; 3: bulliform; 4: irregular; 5: echinate (elongate dentate); 6: dendritic (elongate dendritic); 7: discoid; 8: spheroid echinate; 9: trapeziform (trapezoid). Note the carbonized organic material dominating sample 4 from Nessana (C).

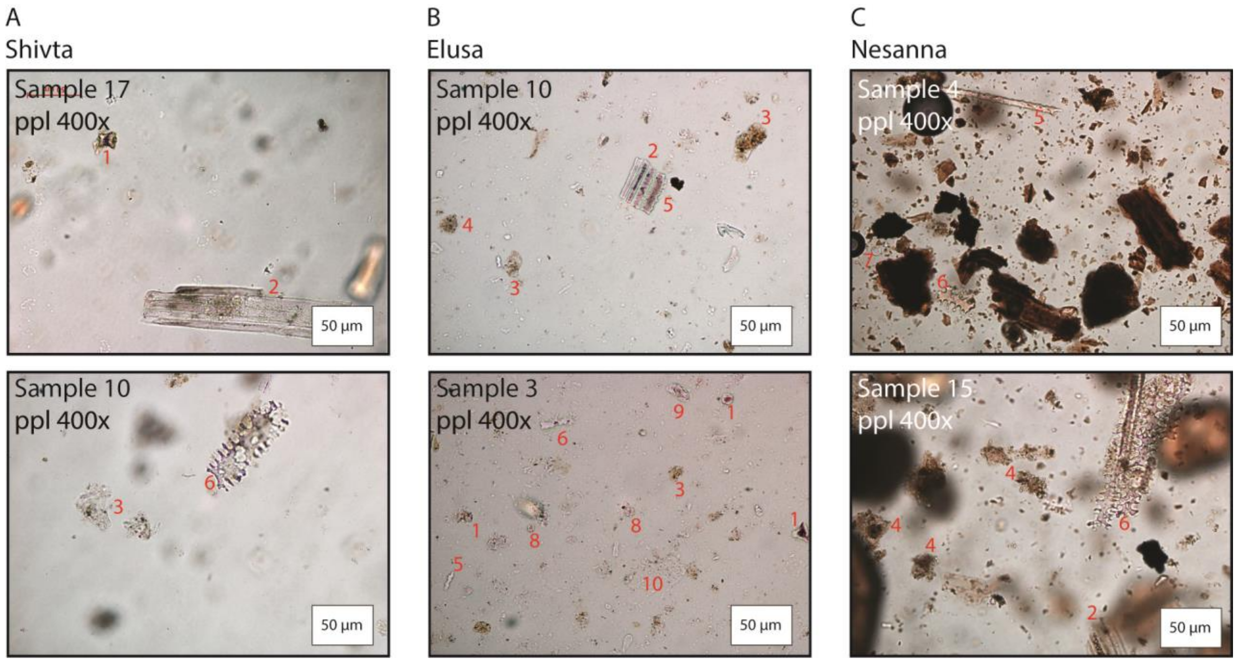

Results of the phytolith morphotype analyses are presented in S2 Fig. At Shivta, the soft dark brown/grey sediment (sample 10) was dominated by wild grasses. Grass leaves, stems, and inflorescence were all represented. The other layer types at Shivta contained high concentrations of dicot leaf and wood/bark morphotypes (samples 3, 4, 13, and 17). The grey ashy lens (sample 17), in specific, contained high concentrations of wood/bark phytoliths. We also identified a small amount of spheroid echinate phytoliths indicative of palm in the light brown-yellowish gravel deposit (sample 4).

The lighter colored gravel rich sediments (samples 3 and 10) at Elusa contained relatively high concentrations of wood/bark phytoliths and spheroid echinates from palm. Two of the darker grey layers (samples 2 and 13) also had high quantities of wood/bark morphotypes. Grass phytolith assemblages from the darker grey layers deeper within the profile (samples 2, 5, and 7) were characterized by high proportions of domesticates as well as strong inflorescence representation (i.e., chaff/threshing wastes). In contrast, whole wild grasses and wood were well represented in the darker layers toward the top of the profile section (sample 13).

Assemblages from the Byzantine component at Nessana (samples 24 - 26) contained internally consistent quantities of grass and wood phytoliths. Grass assemblages were primarily characterized by large amounts of inflorescence (i.e., chaff/threshing wastes). Sediments from the Early Islamic component (samples 12, 15, 18, and 19) also had uniform assemblages dominated by grass morphotypes with strong tendencies toward inflorescence. Domesticated grasses dominated samples 12 and 19, yet wild grasses were more prevalent in samples 15 and 18.

S2 Table. Comparison of representative phytolith morphotype characteristics by sample. Percentages of multicells, weathered morphotypes, and dendritics along with the grass leaf/stem to grass influence ratio are displayed. Abbreviations: Byz. = Byzantine; Isl.= Early Islamic; Multi.= multicell phytoliths; Wth. = weathered phytoliths; Den. = dendritic phytoliths; GrL. : GrI. = grass leaves/stems to grass inflorescence ratio; dom: domesticated; inf = tendency toward inflorescence; inf.s.: inflorescence selection; wh = whole.

Site	Period	Sample	Multi (%)	Wth. (%)	Dend. (%)	GrL:GrI
**Shivta**	Byz.	3	4	64	1 (wild)	0.6 (inf.)
	Byz.	4	3	46	1 (wild)	1 (inf.)
	Byz.	10	46	10	6 (wild)	2 (wh.)
	Byz.	13	2	17	-	1.2 (wh.)
	Byz.	17	11	13	-	2 (wh.)
**Elusa**	Byz.	3	0	63	1 (wild)	1 (inf.)
	Byz.	10	2	48	-	3 (wh.)
	Byz	2	3	20	13 (dom.)	0.1(inf.s.)
	Byz	5	41	4	9 (dom.)	0.2 (inf.s.)
	Byz	7	33	13	12 (dom.)	0.1(inf.s.)
	Byz	11	20	27	3 (wild)	2 (wh.)
	Byz.	13	38	29	3 (wild)	1.2 (wh.)
**Nessana**	Isl.	12	4	15	9 (dom.)	0.3 (inf.)
	Isl.	18	7	2	1 (wild)	0.2 (inf.s.)
	Isl.	19	3	36	10 (dom.)	0.2 (inf.)
	Isl.	15	0	0	6 (wild)	0.4 (inf.s.)
	Isl.	20	4	14	-	0.2 (inf.s.)
	Byz.	26	7	6	1 (wild)	0.3 (inf.s.)
	Byz.	25	8	10	2 (wild)	0.1 (inf.s.)
	Byz.	24	5	8	10 (dom.)	0.1 (inf.s.)

S2 Fig. Quantities of representative phytolith morphotypes recorded in the Shivta, Elusa, and Nessana hinterland trash mound sediments. Percentages of phytoliths indicative of grass leaves/stems, grass leaves/stems or inflorescence, grass inflorescence, dicot leaves, dicot wood/bark, palm, and indeterminate sources are shown. Analyzed samples are marked by asterisks in Fig 2. Two hundred phytoliths could not be counted in sample 4 from Shivta (n = 83).

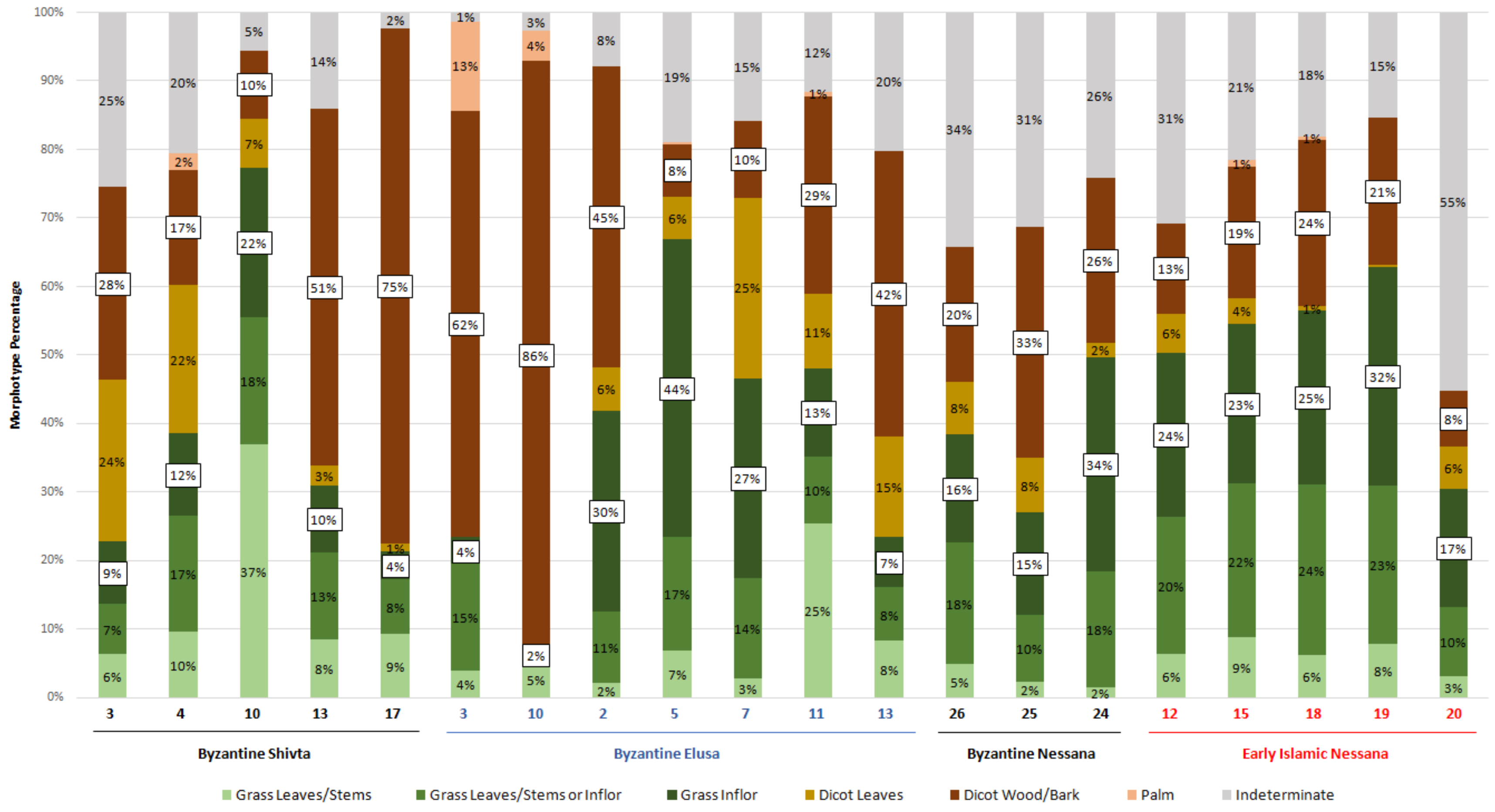

B. Calcitic micro-remains

Roughly 20 - 30 mg of sediment was passed through a 150 µm sieve, reweighed, and placed in a 0.5 ml Eppendorf centrifuge tube. Five hundred µl of SPT was added, and the mixture was vortexed, then sonicated for 10 min. The sample was vortexed again and 50 µl was immediately slide mounted under a 24 × 24 mm cover slip. Counting and calculation procedures are described in the main text. Examples of identified ash pseudomorphs and dung spherulites are provided in S3 Fig. Ovicaprine dung-dominated ash typically has PSR values below 1, while wood-dominated fuel produces ratios greater than 5 [63, 73]. Dung-dominated fuels may also contain large quantities of grass phytoliths, or varying quantities of grass and wood phytoliths, depending on their diets [15]. Unashed samples were also explored for the presence of heated dung spherulites, which are typically characterized by darkened, isotropic centers and distorted shapes when viewed under ppl. Spherulite darkening can occur at temperatures of 500 °C - 600 °C in organic rich, oxygen deprived conditions [83].

Calcitic ash pseudomorphs and dung spherulites will dissolve under mildly acidic, wet conditions [63, 73]. We identified the pH of the sediments to ensure that any discovered patterns were not caused by differential diagenesis. Measurements were taken from slurries of 5 g of unashed sediment and 50 ml of calcium chloride solution using a Jenway 3540 pH meter.

S3 Fig. Representative calcitic micro-remains. (A) Byzantine Shivta. Low quantities of dung spherulites were identified. The largest quantities of ash pseudomorphs in the Byzantine layers studied at Shivta were identified in samples 16 and 17 from an ashy lens. (B) Byzantine Elusa. Low quantities of dung spherulites were also identified at Elusa. Elusa displayed the highest concentrations of ash pseudomorphs. (C) Nessana. Large clusters of dung spherulites and heated spherulites were identified exclusively in Early Islamic samples from Nessana.

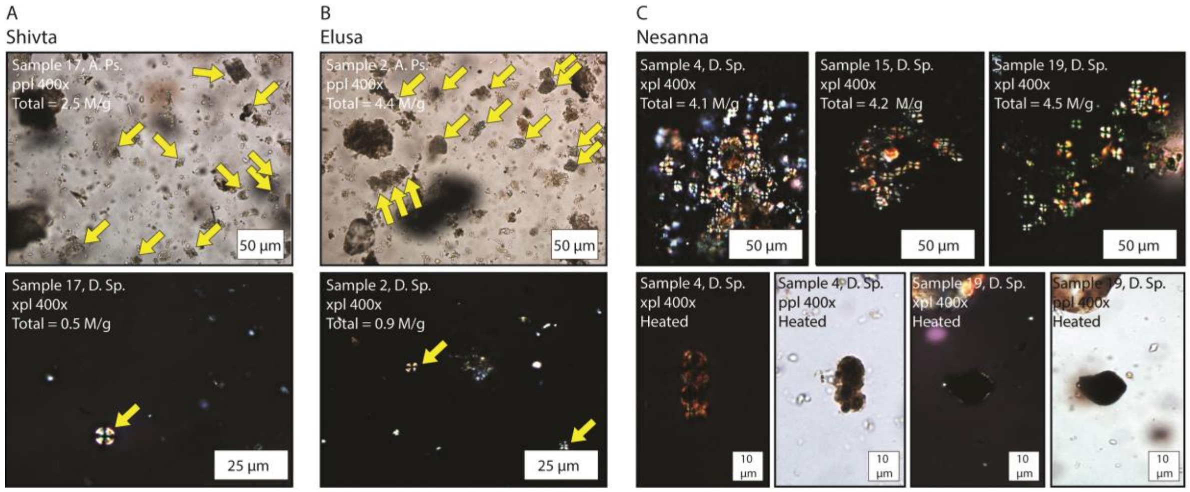

C. FTIR analyses

A roughly 1:20 ratio of sediment fine fraction to potassium bromide was ground and pressed into a pellet. Spectra were averaged from 32 scans in the 4,000 to 400 cm^{-1} range at 4 cm^{-1} resolution. Geogenic and pyrogenic calcites (limestone, chalk v. ash, plaster) were distinguished in FTIR spectra using the approach detailed in Regev et al. [70], which focuses on identifying order/disorder in the atomic structures of calcite ($CaCO_3$) based on the characteristics of the absorbance bands at 1420, 875, and 713 cm^{-1} (all C-O). Aragonite ($CaCO_3$) may also form at temperatures above 600 °C, indicated by the appearance of key IR bands at 1,430, 856, and 700 cm^{-1} (all C-O) [80].

Clay minerals (e.g., montmorillonite; $Na_{0.2}Ca_{0.1}Al_2Si_4O_{10}(OH)_2(H_2O)_{10}$) were identified using diagnostic IR absorbance bands at 1035 (Si-O-Si), 3697, 3620, and 915 cm^{-1} (hydroxyl; OH), and at 525 and 470 cm^{-1} (Si-O-Al and Si-O respectively) [69]. Heat altered clay minerals were distinguished using the method developed by Berna et al. [69]. Generally, clays heated at temperatures of 500 - 600 °C are dehydrated, represented by losses of OH absorbance bands. They also display increasing disorder in the mineral crystal structure, indicated by a leftward shift in the 1035 cm^{-1} band to roughly 1040 - 1050 cm^{-1}, a broadening of this band, and a reduction in the split between the 525 and 470 cm^{-1} bands. Clay heated at roughly 700 °C displays a further leftward shift and broadening of the 1035 cm^{-1} band, as well as the closure of the split between the 525 and 470 cm^{-1} bands. A shift in the 1035 cm^{-1} band as far left as 1090 cm^{-1} is consistent with exposure to temperatures exceeding 800 °C.

We also explored trash mound sediments for the presence of anhydrite ($CaSO_4$), gypsum ($CaSO_4 \cdot 2H_2O$), and carbonated hydroxylapatite (CHAP; $Ca_{10}(PO4)_6(CO_3)_x(OH)_{2-2x}$) to improve our understanding of burning episodes [63, 73, 80]. Anhydrite has key IR bands at 1154, 1125, 676, 613, and 595 cm^{-1} (S-O). This mineral is a component of ash produced by tamarisk, but it transforms into gypsum upon hydration with water. Gypsum displays diagnostic IR bands at 3540, 3400, 1620, 1143, 1117, 671, and 602 cm^{-1} (S-O). These minerals may indicate the presence of wood ash produced from tamarisk, yet gypsum is also commonly found as secondary pedogenic nodules and crusts in arid locations [15]. The CHAP mineral displays key absorbance bands for carbonate at 1420 and 875 cm^{-1} (C-O), and phosphate at 1035, 961, 606, and 565 cm^{-1} (P-O). The mineral may indicate the recrystallization of decomposing calcitic ash. Mixtures of atomically disordered calcite, gypsum, and CHAP are also typical of ashes produced from mixed wood/dung fuel sources [63, 73].

S4 Fig. Representative infrared spectra from the sampled trash mound sediments. These spectra show the presence of unaltered clay, heat altered clay, geogenic calcite, ash calcite, carbonated hydroxylapatite, gypsum, opal, and aragonite. Note that samples from Early Islamic Nessana (C) have high dung spherulite and high sodium nitrate contents, while samples from Byzantine Shivta (A) and Byzantine Elusa (B) have low dung spherulite and sodium nitrate contents.

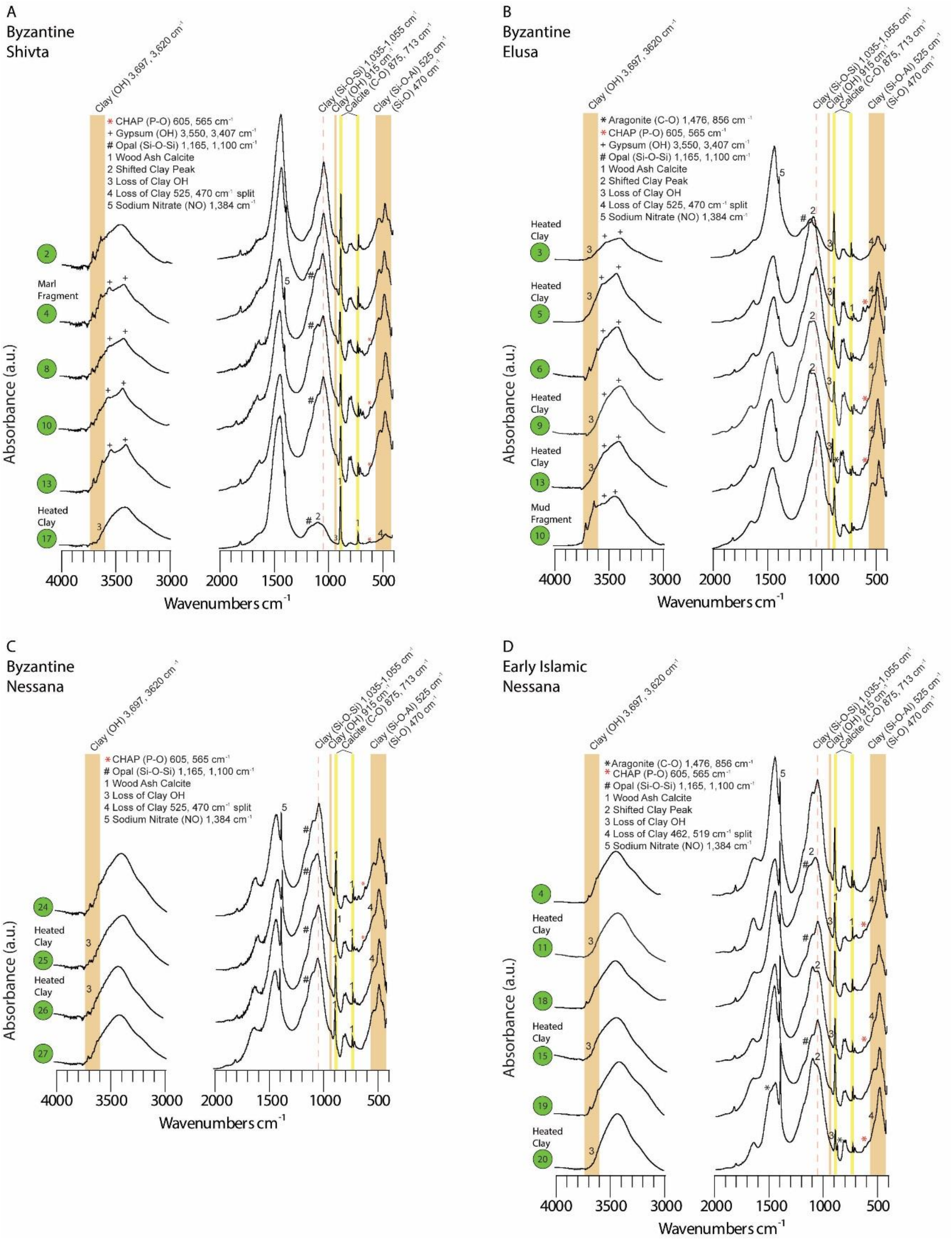

D. Reference materials

S5 Fig . Reference sample locations.

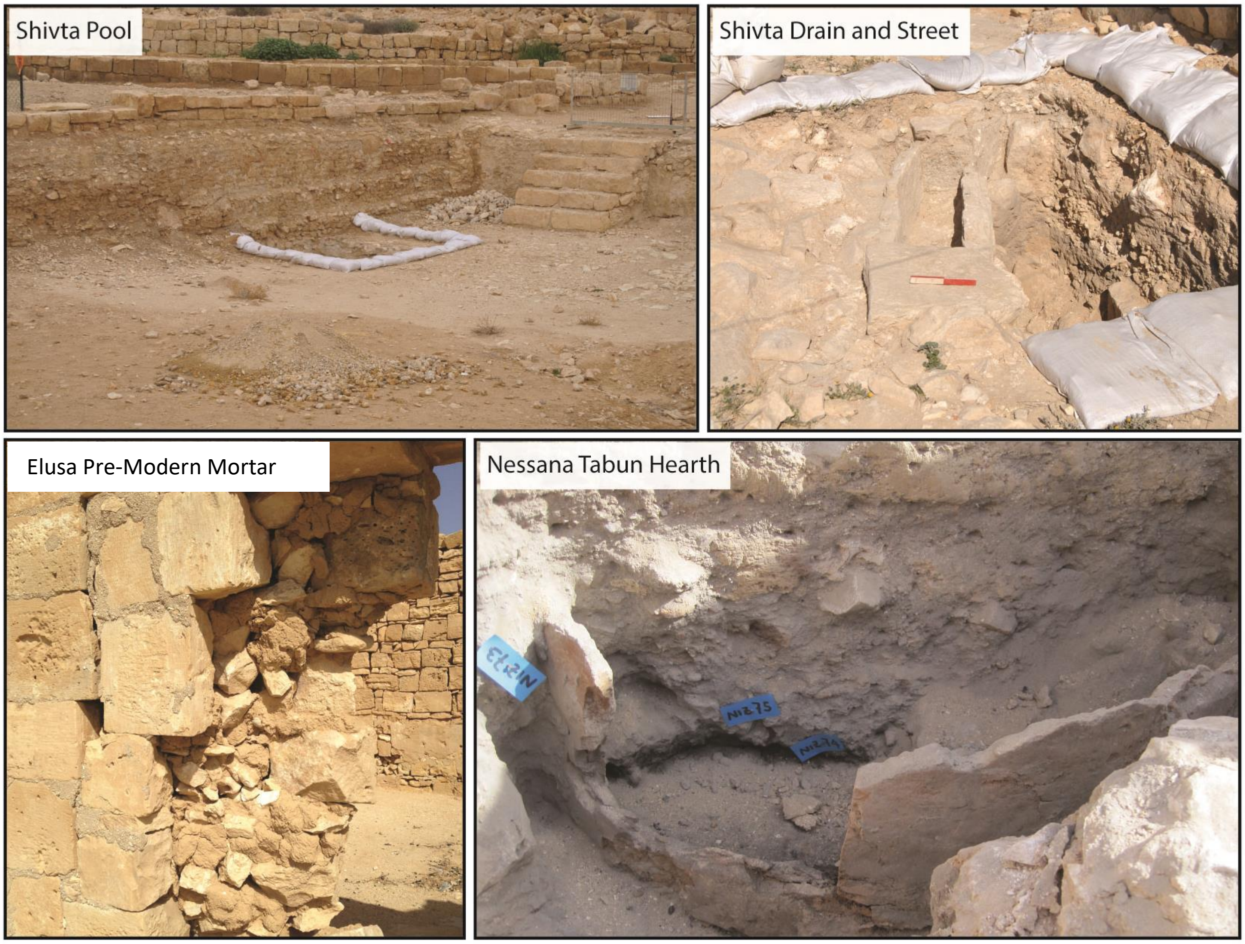

S6 Fig. Infrared spectra of reference materials.

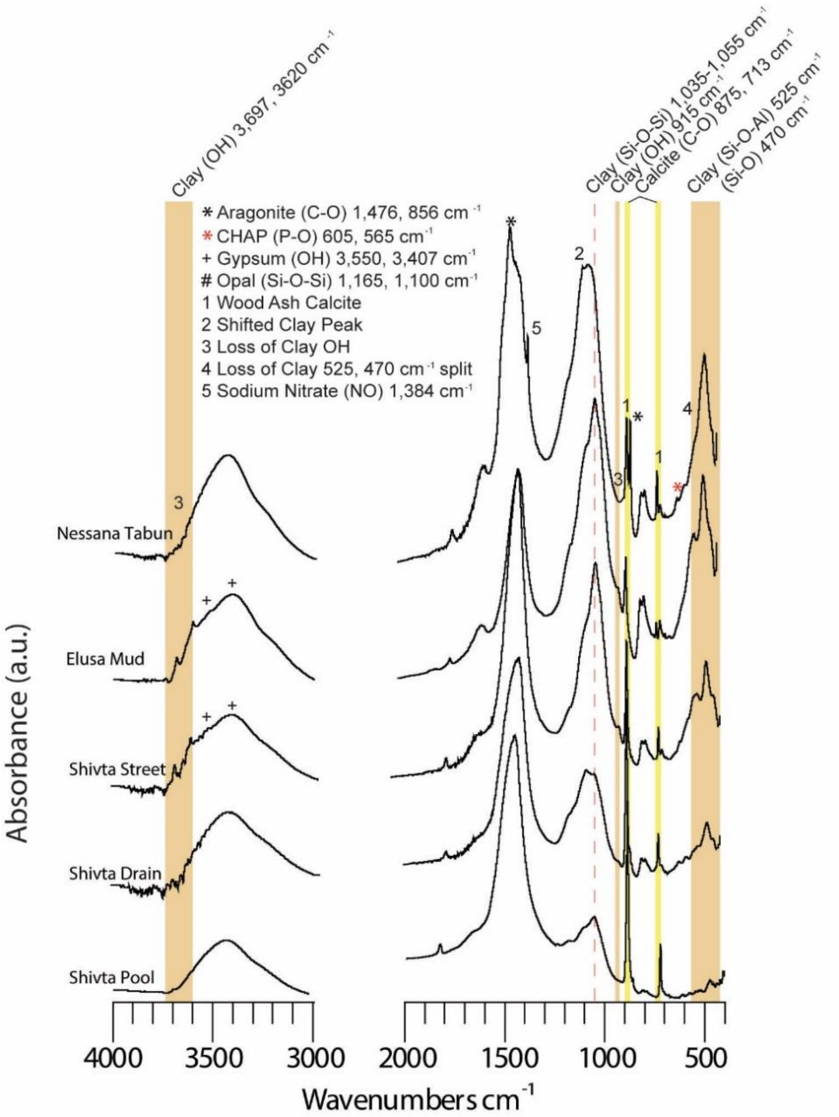

S3 Table. Results for reference samples. Abbreviations: M/g = millions of micro-remains per gram of sediment; PY = phytoliths; DS = dung spherulites; AP = ash pseudomorphs; PY, DS, and AP are presented in millions of micro-remains per gram of sediment; PSR = ash pseudomorph to dung spherulite ratio; OM = organic matter presented in weight percent; Cy (ua/500 °C) = clay (unaltered/heat altered at the designated temperature); Cl (g/a/p) = calcite (geogenic/ash/plaster); Q = quartz; G= gypsum; N = sodium nitrate; O = opal; Ap = apatite; Ar = aragonite; An = anhydrite; ND = no data.

Site	Period	Sample Locaton	PY	DS	AP	PSR	OM	Mineralogy
Shivta	Modern	Control Sed.	0	0.07± 0.03	0	0	7± 3	Cy (ua), Cl (g), Q
Shivta	Byzantine	Drain System	3	0.4	0	0	10	Cy (ua), Cl (p), Q, Ap
Shivta	Byzantine	Pool	2	0.1	0.1	1	9	Cy (ua), Cl (g), Q
Shivta	Byzantine	Street	0.4	0.7	0.6	0.6	8	Cy (ua), Cl (g), Q, G
Elusa	Modern	Mud Morter	ND	ND	ND	ND	ND	Cy (ua), Cl (g), Q, G
Nessana	Byzantine	Tabun Hearth	15	18	8	0.4	6	Cy (600-700°C), Cl(a), Q, G, N, Ar, Ap
