# Supplementary material for: Byzantine—Early Islamic resource management detected through micro-geoarchaeological investigations of trash mounds (Negev, Israel)
Source: PLoS One. 2020 Oct 14;15(10):e0239227. doi: 10.1371/journal.pone.0239227 (PMC7556535; doi:10.1371/journal.pone.0239227)
Supplement: S2 File — (PDF) [file pone.0239227.s002.pdf]

Request for Permission to Publish Content under CC-BY License

Dear Rights Holder or Representative,

I have submitted a paper for publication in a PLOS journal, and wish to include the content listed below in the paper. I'm hereby requesting your (or your company's or institution's) permission to include the content in my paper. Please note that all PLOS journals are published under a Creative Commons Attribution License (CC BY), which allows for unrestricted use and distribution, even commercial, as long as attribution is given to the creator or rights holder of the content. See https://creativecommons.org/licenses/by/4.0/.

To grant me permission to use the content in my PLOS paper, please fill in the information below and then scan the completed form and send it to me at my email address.

Thank you.

My name:

Don Butler

My email address:

dhbutler3@alaska.edu

Description of the content which I'm seeking permission to use (citation and/or title, and pasted screen shot, if applicable):

Orthophoto images of the archaeological sites of Shivta, Elusa, and Nessana.

Link to the Content:

https://www.govmap.gov.il/?lang=en

* * *

On behalf of myself or the rights holder, I hereby grant the permission sought herein.

Signature of Party Granting Permission:

Date:

20/08/2020

Printed Name and Title:

Iris Tziha
GIS customer services manager
Division of Policy and Service Quality

August 8th, 2020
Mr. Ronen Regev
Director General of the Survey of Israel

Dear Mr. Regev and persons acting on his behalf,

We, Don Butler and Ruth Shahack-Gross, are researchers at the University of Haifa Department of Maritime Civilizations. We write to request written permission for the open-access journal PLOS ONE to publish images from govmap.gov.il under the Creative Commons Attribution License (CCAL) CC BY 4.0. We ask to please use images of the archaeological sites of Shivta, Elusa, and Nessana in our work. Please find the images presented below.

I, Don Butler, am currently in the United States, and can be reached by phone at +1-907-251-9055 or email dhbutler@ucalgary.ca.

Thank you for your time and consideration,

Sincerely,

Don H. Butler

Department of Maritime Civilizations
University of Haifa
199 Abba Khoushy Ave.
Haifa, Israel, 3498838
dhbutler@ucalgary.ca
+1-907-251-9055

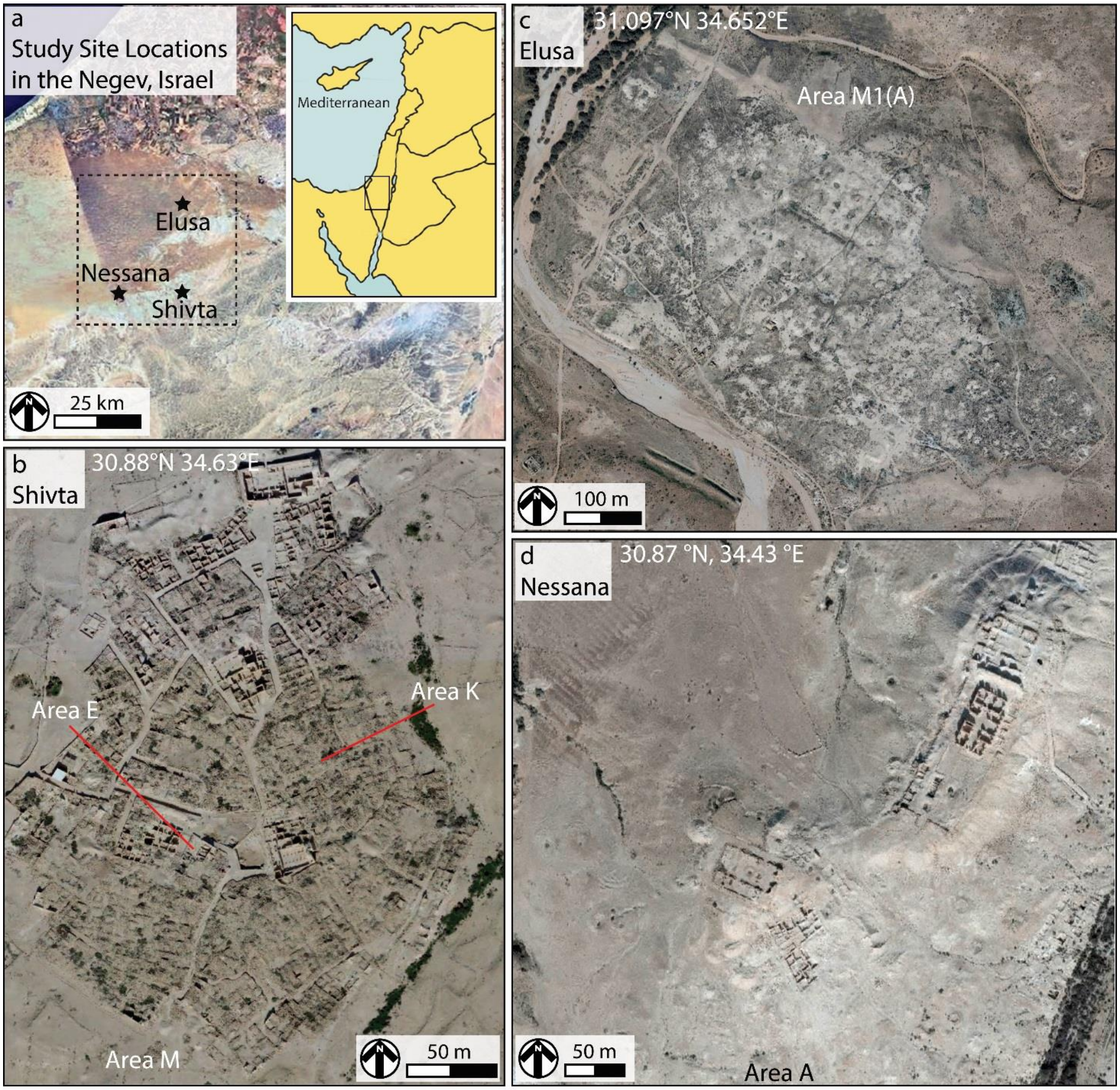
a
Study Site Locations in the Negev, Israel
Mediterranean
Elusa
Nessana
Shivta
25 km
b
Shivta
30.88°N 34.63°E
Area E
Area K
Area M
50 m
c
Elusa
31.097°N 34.652°E
Area M1(A)
100 m
d
Nessana
30.87 °N, 34.43 °E
Area A
50 m

permission to publish orthophoto images from govmap

שירות לקוחות <customerservice@mapi.gov.il>
Mon 8/10/2020 4:13 AM
To: Donald Butler <dhbutler@ucalgary.ca>
Cc: רונן רגב <ronenr@mapi.gov.il>; אלקנה דשן <elkanad@mapi.gov.il>; רונית טרבלוס-ממוקה <ronitt@mapi.gov.il>

1 attachments (415 KB)
FaxMessage.pdf;

[△EXTERNAL]

Dear Don Butler,

The Survey of Israel hereby grants you permission to publish orthophoto images from govmap.gov.il of archaeological sites of Shivta, Elusa and Nessana in your research work, which will be published in PLOS ONE journal.

The permission for this purpose is granted with no charge.

Any other uses of those orthophoto images will require an additional written agreement from the Survey of Israel.

The images have to include this sub-title and logos.

©2020 All Rights Reserved by the Survey of Israel
Published with Survey of Israel permission.

Please let us know when your research will be published by send us a link of it.

Good Luck

Best Regards,

Iris Tziha
GIS customer services manager

Division of Policy and Service Quality
Tel': +97236231813
www.mapi.gov.il
